# Crystal structures of a halophilic archaeal malate synthase from *Haloferax volcanii *and comparisons with isoforms A and G

**DOI:** 10.1186/1472-6807-11-23

**Published:** 2011-05-10

**Authors:** Colten D Bracken, Amber M Neighbor, Kenneth K Lamlenn, Geoffrey C Thomas, Heidi L Schubert, Frank G Whitby, Bruce R Howard

**Affiliations:** 1 Department of Physical Science, Southern Utah University, Cedar City, UT 84720-2470, USA; 2 Department of Biochemistry, University of Utah, Salt Lake City, UT 84112-5650, USA; 3 Department of Pharmacology, Georgetown University Medical Center, Washington, DC. 20057-1411, USA; 4 Department of Chemistry, University of Utah, Salt Lake City, UT 84112-0850, USA

## Abstract

**Background:**

Malate synthase, one of the two enzymes unique to the glyoxylate cycle, is found in all three domains of life, and is crucial to the utilization of two-carbon compounds for net biosynthetic pathways such as gluconeogenesis. In addition to the main isoforms A and G, so named because of their differential expression in *E. coli *grown on either acetate or glycolate respectively, a third distinct isoform has been identified. These three isoforms differ considerably in size and sequence conservation. The A isoform (MSA) comprises ~530 residues, the G isoform (MSG) is ~730 residues, and this third isoform (MSH-halophilic) is ~430 residues in length. Both isoforms A and G have been structurally characterized in detail, but no structures have been reported for the H isoform which has been found thus far only in members of the halophilic Archaea.

**Results:**

We have solved the structure of a malate synthase H (MSH) isoform member from *Haloferax volcanii *in complex with glyoxylate at 2.51 Å resolution, and also as a ternary complex with acetyl-coenzyme A and pyruvate at 1.95 Å. Like the A and G isoforms, MSH is based on a β8/α8 (TIM) barrel. Unlike previously solved malate synthase structures which are all monomeric, this enzyme is found in the native state as a trimer/hexamer equilibrium. Compared to isoforms A and G, MSH displays deletion of an N-terminal domain and a smaller deletion at the C-terminus. The MSH active site is closely superimposable with those of MSA and MSG, with the ternary complex indicating a nucleophilic attack on pyruvate by the enolate intermediate of acetyl-coenzyme A.

**Conclusions:**

The reported structures of MSH from *Haloferax volcanii *allow a detailed analysis and comparison with previously solved structures of isoforms A and G. These structural comparisons provide insight into evolutionary relationships among these isoforms, and also indicate that despite the size and sequence variation, and the truncated C-terminal domain of the H isoform, the catalytic mechanism is conserved. Sequence analysis in light of the structure indicates that additional members of isoform H likely exist in the databases but have been misannotated.

## Background

The glyoxylate cycle, originally described by Kornberg and Krebs [1], is essential for microorganisms surviving on two-carbon compounds as sole carbon sources. A variant on the tricarboxylic acid cycle (TCA), it allows conversion of two-carbon compounds such as acetate into TCA cycle intermediates, to supply necessary metabolite building blocks such as amino acids and carbohydrates. Two enzymes, isocitrate lyase and malate synthase are unique to the glyoxylate cycle. First, isocitrate lyase cleaves isocitrate to form succinate and glyoxylate, thereby bypassing steps in the TCA cycle that would normally evolve two molecules of CO_2_. These two carbon atoms instead are maintained as the two-carbon compound glyoxylate, which can then react in a Claison condensation with acetyl-coenzyme A (acetyl-CoA) to form a malyl-CoA intermediate that is subsequently hydrolyzed to produce malate and CoA. This condensation and subsequent hydrolysis are catalyzed by malate synthase. Thus the glyoxylate cycle allows the conversion of one TCA cycle intermediate to two, using two acetyl groups from CoA to form the second. This pathway therefore allows organisms to utilize acetyl groups for net biosynthesis such as in the conversion of oils stored within plant seeds to carbohydrates for the construction of plant tissues during germination. Importantly, the glyoxylate cycle has been shown to contribute to the virulence of several human pathogens including *Mycobacterium tuberculosis *[2] and *Candida albicans *[3], and its absence in humans makes it an attractive target for the development of novel antibacterial and antifungal drugs [4,5]. Interestingly, this pathway has recently been implicated in the process of fruit ripening [6].

Malate synthase activity was initially discovered in *E. coli *[7]. Since then it has been found in a wide range of organisms including many bacteria, plants, and fungi; and even in some animals. Although there is a report that gene sequences coding for malate synthase have been identified in the genome sequences of platypus and opossum [8], a UniProt database search [9] shows that there currently are no malate synthase sequences deposited for any reptiles, birds or mammals. While it has been long appreciated that the glyoxylate cycle is distributed widely in bacteria and eukaryotic organisms, it wasn't until recently that it became clear this metabolic pathway is also found in the domain Archaea [10], and therefore spans all three domains of life.

There are two main isoforms of malate synthase: MSA and MSG, originally identified in *E. coli *grown on either acetate (A) or glycolate (G) respectively [11,12]. These two isoforms differ significantly in both size and sequence homology. Members of isoform A comprise ~530 amino acid residues, while those belonging to isoform G comprise ~730 residues. Although the sequence conservation among MSA isoform members, and among MSG members is high (27-99% and 49-98% sequence identity respectively), the sequence identity for structurally conserved regions between these two isoforms is only ~18% [4]. More recently, two examples of malate synthase representing novel isoforms have been found in Archaea [10,13]. The first example of an archaeal malate synthase was purified from *Haloferax volcanii *[10], a halophile originally isolated from the mud of the Dead Sea [14]. This malate synthase, encoded by the *aceB *gene, comprises only 433 residues, shares very little sequence identity with either the A or G isoform (estimated at 10.2-14.1% and 10.5-12.0% respectively) [15], and therefore belongs to a third isoform of this enzyme. A BLAST search against the current UniProt database using the *H. volcanii *sequence as a query indicates a 23% identity with some MSA members found in bacteria of the order actinomycetales, suggesting a closer relationship with the MSA isoform than previously thought. Other examples of isoform H have been identified in genome sequencing projects of halophilic Archaea, including an additional variant in *H. volcanii *[16,17]. Since this new isoform is found thus far only in halophilic archaeal organisms, it has been proposed to denote it as isoform H (MSH) [18], a convention we will continue to use here for comparisons with the other two well-characterized isoforms MSA and MSG.

Comparison of the H isoform with MSA and MSG offers potential insight into the adaptation of this enzyme to a high-salt environment such as found in the Dead Sea. Halophilic archaea have been shown to accumulate KCl to concentrations as high as 4.2 M in order to maintain turgor pressure in such an environment [19,20]. Proteins within organisms like *Haloferax volcanii *have acquired characteristics that allow them to be soluble, stable and functional at these high ionic strengths. *H. volcanii *MSH, for example, displays optimal activity in 3 M KCl [10], which is similar to levels expected in vivo [20]. One common characteristic of proteins functioning in these high-salt environments is a drastic increase in the number of acidic residues, especially aspartate, and a corresponding decrease in lysine [21-23]. Other characteristics have been described including a decrease in overall hydrophobic content [24-26], increased ion binding [27], ordered water networks and intermolecular ion pairs [28-30]. Although much attention has focused on the role of increased surface acidity in protein stabilization due to increased binding of water and ions, a recent study in which surface residues of an obligate halophilic protein were systematically mutated to convert it to a non-halophilic protein and also the reverse, indicated that overall protein charge was not vital [31]. Rather it was concluded that halophilicity is directly related to a decrease in the solvent accessible surface. It has been proposed that the increase in aspartate and decrease in lysine residues may be the result of genetic drift with the increased GC content of genomic DNA in halophilic organisms [22]. However, the high GC content among halophiles is not universal [26,32], and reshuffling of halophilic proteomes at the DNA level demonstrates that the amino acid bias found in halophiles is not a consequence of mononucleotide composition bias [26].

A fourth isoform of malate synthase has been found in crenarchaeal species which is approximately 100 residues larger than the MSG isoform and shares only low levels of sequence identity with the other three isoforms of malate synthase. The *Sulfolobus acidocaldarius *malate synthase, for example, is composed of 824 residues and shares only 31% identity with *E. coli *MSA [13,33]. Intriguingly, there is no magnesium requirement for catalytic activity of this fourth isoform, and it therefore may function via a mechanism distinct from the other three isoforms [13].

Structure determinations of MSG [34-37] and MSA [4] by X-ray crystallography and MSG by nuclear magnetic resonance [38] have revealed structural and functional similarities and differences. While previously solved structures for MSA and MSG have revealed monomeric enzymes, MSH from *H. volcanii *has been reported to exist in the native state as a trimer [10], but was later revised to a tetramer [15]. In order to understand how MSH relates structurally to the larger MSA and MSG isoforms; to clarify the native oligomeric state and understand how it relates to previously solved monomeric versions, and to gain insight into its mechanisms of haloadaptation, we have determined crystal structures of *H. volcanii *malate synthase in complex with glyoxylate, and also as a ternary complex with acetyl-coenzyme A and pyruvate.

## Results and Discussion

### *H. volcanii *MSH Structure

*Haloferax volcanii *malate synthase crystallized in the rhombohedral space group R32 with one monomer per asymmetric unit. The structure was solved with the SIRAS method (single isomorphous replacement with anomalous scattering) using a native dataset collected to 2.7 Å, and a lead derivative diffracting to 2.1 Å resolution, both in the presence of 3 mM glyoxylate (Table 1). An atomic model was built manually into the experimental map, and was used for molecular replacement to solve the high-occupancy glyoxylate complex at 2.51 Å, and the pyruvate/acetyl-CoA complex.

**Table 1:** Crystallographic Data and Refinement Statistics

	**Native**^**a**^	Pb Derivative	Glyoxylate Complex	Ternary Complex
**Data Collection**				
Unit cell dimensions				
a = b (Å)	156.4	155.4	155.0	154.8
c (Å)	141.5	139.5	141.8	142.1
α = β (°)	90	90	90	90
γ (°)	120	120	120	120
Resolution (Å)	30-2.70	30-2.10	20-2.51	30-1.95
	(2.80-2.70)	(2.18-2.10)	(2.59-2.51)	(2.02-1.95)
Number of observations				
Total	104,473	271,479	75,347	401,261
Unique	18,255	73,241^b^	22,203	47,508
Redundancy	5.7 (5.7)	3.7 (3.6)^b^	3.4 (3.2)	8.4 (7.8)
Complete (%)	100.0 (100.0)	99.5 (100.0)	98.8 (97.3)	100.0 (100.0)
R_sym_^c^	0.090 (0.383)	0.103 (0.374)	0.096 (0.453)	0.099 (0.806)
<I/σ(I)>	14.2 (4.2)	11.5 (3.3)	8.7 (2.5)	15.2 (2.3)
Wilson B factor (Å^2^)	68.6	33.6	56.0	33.5
Figure of Merit from SOLVE		0.36		
Figure of Merit after RESOLVE		0.71		
Cullis R factor		0.74		
R_iso_/R_ano_		0.285/0.054		
**Refinement**				
R_work_^d^	0.1984		0.1921	0.2029
R_free_^e^	0.2626		0.2476	0.2390
R.m.s. deviations				
Bond lengths (Å)	0.014		0.017	0.017
Bond angles (°)	2.27		2.07	2.47
Mean isotropic B factor (Å^2^)	58.34		58.33	47.65
Φ/Ψ angles^f^				
Most favored (%)	89		86.3	91.8
Additional allowed (%)	11		13	7.9
Generously allowed (%)	0		0.3 (Thr 276)	0.3 (Glu 24)
Disallowed (%)	0		0.3 (Glu 24)	0

Values in parentheses are for the high-resolution shell. ^a^At 3 mM glyoxylate, 13 mM Mg^2+^. ^b^Friedel mates treated as independent reflections for anomalous phasing. ^c^R_sym _= Σ_hkl _|I - <I>|/Σ_hkl _(I), where I is the observed intensity, and <I> is the average intensity for multiple observations of symmetry related reflections. ^d^R_work _is the R_factor _for 95% of data used during refinement, where R_factor _= Σ_hkl _||F_o_|-|F_c_||/Σ_hkl_|F_o_|. ^e^R_free _is the R_factor _for 5% of the data not used in refinement. ^f^For non-Gly and non-Pro residues only.

Two lead binding sites were used for phasing, and a third lower-occupancy site was identified during model building and refinement of the lead derivative. Two of the sites were found at or near intersubunit interfaces, which may explain the higher-resolution diffraction of the derivative. Unfortunately, lead substitution of the required magnesium ion within the active site precluded its use as a higher-resolution pseudo-native structure.

The native structure (3 mM glyoxylate) was refined to an R_factor _of 0.202 and an R_free _of 0.263. Two substantial loops, residues 283-330 and 355-386 were not ordered in the crystal and have not been included in the refined model, which comprises residues 2-22, 25-282, 331-354, and 387-432, one glyoxylate molecule, one magnesium ion, four potassium ions, four chloride ions, and 71 water molecules. The stereochemistry is satisfactory with no residues in the generously allowed or disallowed regions of the Ramachandran plot (Table 1). Distorted magnesium coordination geometry, apparently caused by binding of an adjacent potassium ion, coupled with high B-factors for Mg^2+ ^and glyoxylate (78 and 72-79 respectively), prompted us to modify conditions to obtain a high-occupancy glyoxylate complex.

The high-occupancy glyoxylate complex was prepared by increasing the concentrations of MgCl_2 _from 13 mM to ~0.1 M and glyoxylate from 3 mM to ~0.1 M in mother liquor after crystal growth was complete and one week prior to data collection. The final model has a crystallographic R_factor _of 0.195 and an R_free _of 0.248, and is comprised of residues 5-283, 331-353, and 387-432, two glyoxylate molecules, one magnesium ion, three potassium ions, five chloride ions and 134 water molecules. One glyoxylate molecule is bound to the Mg^2+ ^at the active site and the other is bound weakly in the position at which the acetyl-CoA thioester resides in the ternary complex (below). The structure is in good agreement with expected stereochemistry, with only one residue in the generously allowed region (Thr 276) and one residue in the disallowed region (Glu 24) of the Ramachandran plot (Table 1).

The pyruvate/acetyl-CoA ternary complex was prepared by soaking crystals in mother liquor containing 50 mM MgCl_2_, and supplemented with ~70 mM pyruvate and ~0.15 M acetyl-CoA one week before data collection. This structure was refined to a crystallographic R_factor _of 0.205 and an R_free _of 0.239. One loop not visible in the glyoxylate complex becomes significantly ordered in the pyruvate/acetyl-CoA complex, and the refined model comprises residues 5-284, 328-371, 381-432, one molecule of pyruvate, one molecule of acetyl-CoA, one magnesium ion, three potassium ions, four chloride ions, a phosphate ion and 176 water molecules. There is only one residue, Glu 24, in the generously allowed region of the Ramachandran plot, and none in the disallowed regions (Table 1).

### Monomer Structure

**Figure 1:**
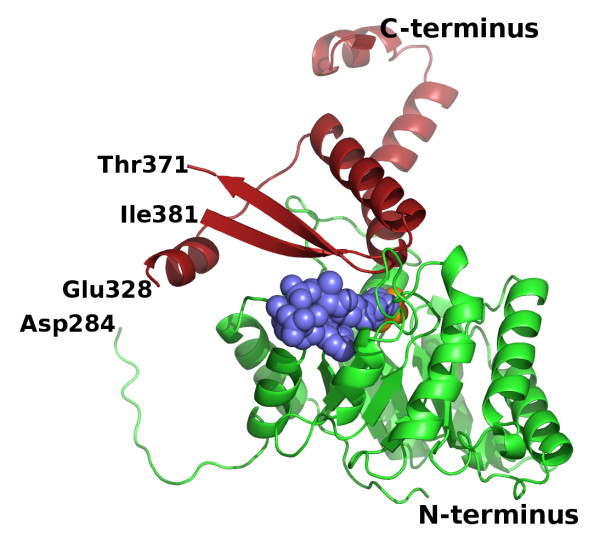
**Overall fold of an *H. volcanii *MSH monomer in the ternary complex**. The N-terminal β8/α8 barrel and the C-terminal domain are shown as cartoon ribbon traces in green and red respectively. Acetyl-coenzyme A and pyruvate are shown as space-filling models in slate blue and orange respectively. The protein segments between Asp284 and Glu328, and between Thr371 and Ile381 were not visible in the crystal structure and have not been included in the model.

The core of *H. volcanii *MSH forms a β8/α8 (TIM) barrel (Figure 1), as observed in MSA and MSG isoforms [4,34,35]. Unlike MSA and MSG, however, the N-terminus of the protein directly precedes the barrel fold, whereas MSA and MSG both have an N-terminal domain that folds against the outside of the barrel, followed by an extended surface loop preceding the start of the first strand of the barrel domain.

**Figure 2:**
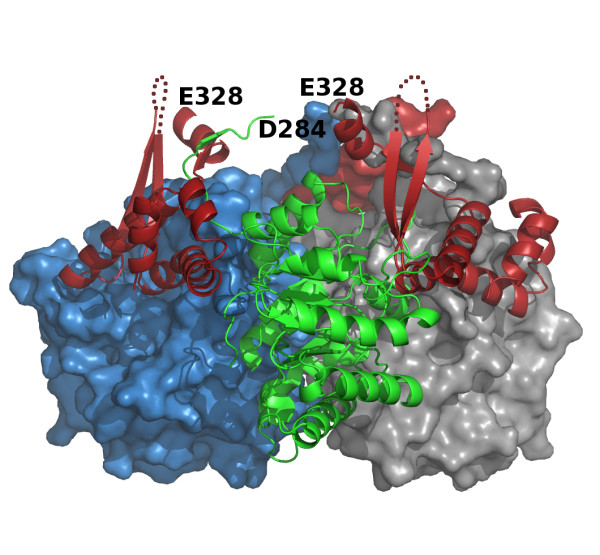
**Possible domain swapping in *H. volcanii *MSH**. The subunit of the trimer closest to the viewer is rendered in the same form and colors as in figure 1, with the C-terminal domain on the right. The C-terminal domain on the left, also rendered as a red cartoon ribbon trace would instead be connected to the green β8/α8 barrel if domain swapping occurs. In the domain swap the C-terminal domain connected to the barrel of one subunit (green) would be donated to complete the active site of the neighboring barrel shown as a blue surface rather than its own. The short disordered surface loops (372-380) are depicted as red dotted lines.

As seen in previously determined malate synthase structures and β8/α8-barrel enzymes in general, the MSH active site is located at the C-terminal ends of the β-strands. The active site is completed by residues from a C-terminal domain of the protein as in MSA and MSG, although the MSH C-terminal domain differs substantially from the other isoforms (below). A break in the electron density (284-330 of glyoxylate and 285-327 of ternary complexes) prevents modeling of the entire connection between the TIM barrel and the C-terminal domain. Seven of these missing 43 residues in the better-resolved ternary structure are glycines, which supports the expectation of considerable flexibility of this region. Due to the length of this missing connection, we cannot eliminate the possibility of domain swapping. Therefore, the C-terminal domain of one subunit may complete the active site by capping its own TIM barrel, as we have modeled it and as it occurs in the other isoforms, or it may cap the TIM barrel of a neighboring subunit. The distance between the backbone carbonyl carbon of Asp 284 and the backbone nitrogen of Glu 328 of the C-terminal domain in our model of the ternary complex is 22.6 Å, while the distance for a domain swap would be 21.3 Å (Figure 2).

### Trimer/Hexamer Structure

The native oligomerization state of hvMSH was initially reported to be a trimer based on gel-filtration mobility and SDS PAGE analysis with estimated molecular weights of 200 ± 30 kDa and 67 ± 4 kDa for the native enzyme and individual subunits respectively [10]. But after the *aceB *gene was cloned, it became clear that individual subunits were actually only 47.9 kDa leading to a revised prediction of a tetrameric assembly [15]. This abnormally slow SDS PAGE mobility is a common characteristic of halophilic proteins which have an excess of acidic residues [39].

**Figure 3:**
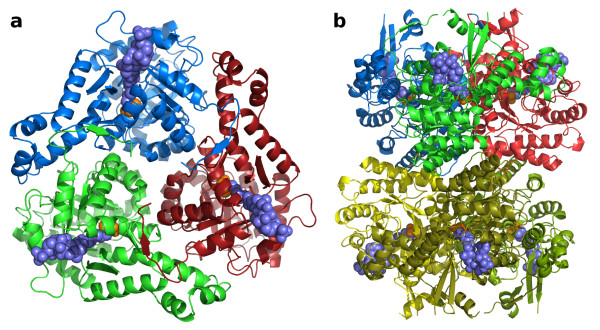
***H. volcanii *MSH oligomerization**. a) A trimeric assembly rendered as cartoon ribbon traces, viewed along a crystallographic 3-fold rotation axis. Acetyl-coenzyme A and pyruvate are shown as space-filling models in slate blue and orange respectively. b) A hexameric assembly viewed along a crystallographic 2-fold rotation axis, perpendicular to the view in part a. The top trimer is rendered and colored as in part a.

Instead of a tetramer, however, a trimer is formed in the hvMSH structure through symmetry operations around a crystallographic three-fold rotation axis. A hexamer is formed by an additional symmetry operation on one trimer around a perpendicular two-fold rotation axis (Figure 3a, b). An analysis of the crystal contacts between monomers using the protein interfaces, surfaces and assemblies service (PISA) at the European Bioinformatics Institute [40] predicts that both the trimeric and hexameric assemblies are thermodynamically stable and biologically relevant. There are approximately 1998 Å^2 ^of buried surface area per subunit at each of the three trimer interfaces. The interface between two trimers that form the hexameric assembly is also substantial: two independent surfaces of approximately 894 Å^2 ^and 258 Å^2^, account for a total of 1152 Å^2 ^buried per subunit, or 3456 Å^2 ^buried per trimer.

**Figure 4:**
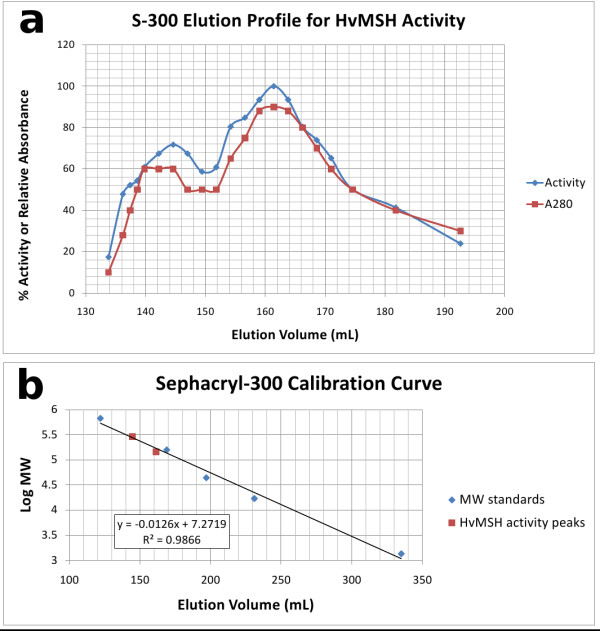
***H. volcanii *MSH Gel-filtration mobility**. a) Gel-filtration elution profile for malate synthase activity and relative absorbance at 280 nm. b) Best-fit linear calibration curve using Bio-Rad gel-filtration standards. Superimposed are the two malate synthase activity peaks, plotted using the elution volume of each and the respective molecular weight of the corresponding hexameric or trimeric assembly.

Both trimeric and hexameric assemblies are also supported by the observed elution profile of purified *H. volcanii *MSH from a Sephacryl-300 gel-filtration column. The elution profile is bimodal, indicating two populations of MSH which differ substantially in hydrodynamic radius (Figure 4a). The elution volume for the first peak of malate synthase catalytic activity is consistent with that expected for a hexamer (logMW = 5.46) (Figure 4). While the elution volume of the second peak falls midway between that expected for a tetramer (logMW = 5.28) and a trimer (logMW = 5.16), it is consistent with a trimeric assembly considering the inherent error of ± 20% in estimates of MW using this technique [41].

### Comparison of *H. volcanii *MSH overall structure with *E. coli *MSA and MSG

Molecular overlays of the hvMSH ternary complex onto the corresponding ternary complexes of ecMSA [PDB:3CV2] [4] and ecMSG [PDB:1P7T] [36] were performed with SSM [42]. The overlays used the entire model for each structure involved, and resulted in 271 residues aligning between hvMSH and ecMSA, with an 18.8% identity and a root-mean-square deviation of 1.90 Å for aligned alpha carbons. A similar number of residues aligned between hvMSH and ecMSG: 262 residues with a 17.2% identity and rmsd = 1.85 Å for structurally equivalent Cα positions.

**Figure 5:**
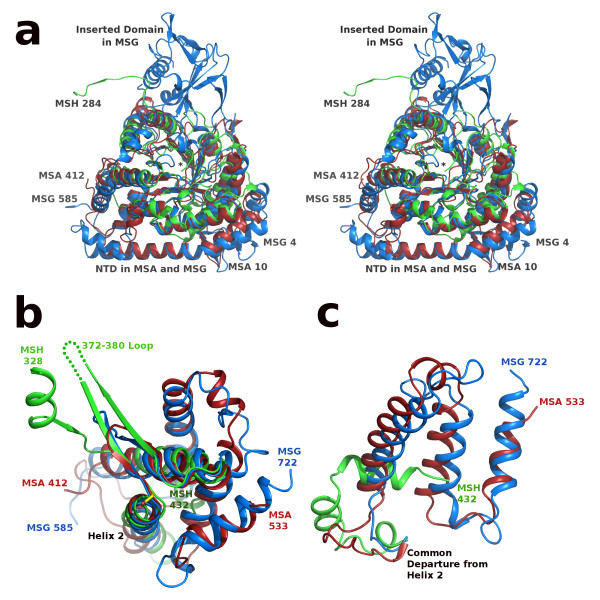
**SSM overlay of MSA [PDB:**3CV2**], MSG [PDB:**1P7T**], and MSH [PDB:**3OYZ**] pyruvate/Acetyl-CoA ternary complexes**. a) Stereoview of the N-terminal regions: MSA 10-412, MSG 4-585 and MSH 5-284 rendered in red, blue and green respectively. Ends of protein chains are labeled with residue numbers except MSH residue 5 which is marked by an asterisk at the center of the image. View is from the bottom of the TIM barrel, opposite the active site; C-terminal domains are not shown for clarity. b) Overlay as in part a, but only showing the C-terminal domains of each protein: MSA 412-533, MSG 585-722, and MSH 328-432. The active site base Asp 388 in MSH is shown (center) in stick form with carbon atoms colored yellow and corresponds to the same position as Asp 447 in MSA and Asp 631 in MSG (within 0.7 and 1.0 Å respectively). Disordered loop in MSH is shown as a green dotted line. c) Overlay as in parts a and b, but only showing extreme C-terminal regions: MSA 463-533, MSG 647-722, and MSH 404-432. Helix 2 refers to the second helix of the C-terminal domain which immediately follows the active site aspartate residue as seen in figure 5b.

The overlays show that the structure of the TIM barrel is conserved among these three isoforms with slight variations (Figure 5a). However, an N-terminal domain which is found preceding the barrel fold in both MSA and MSG structures is missing in MSH. The first strand of the TIM barrel begins with residue 91 in ecMSA, and residue 114 in ecMSG. The structurally equivalent position in hvMSH is residue 12, with the preceding residues forming a short loop that covers the bottom of the barrel. The absence of this extended N-terminal sequence in MSH accounts for an ~80 residue reduction in size compared to the MSA isoform. The overlay also shows that, like MSA, MSH is missing an inserted domain that appears to be found only in MSG (Figure 5a), and is largely responsible for the ~200 residue difference in size between the MSA and MSG isoforms [4].

The ends of the protein segment connecting the TIM barrel and the C-terminal domain that are visible in the hvMSH ternary complex suggest this connection is quite different from those of MSA and MSG. The last structurally equivalent residue in the TIM barrel among these three isoforms is found at the completion of the eighth and final helix: Leu 272, Asn 380, and His 549 in hvMSH, ecMSA and ecMSG respectively. Two of the next three residues in hvMSH are proline (PPK) with the trajectory of the backbone in essentially the opposite direction as those of the comparable segments in MSA and MSG. The next residue that is structurally common to all three is near the beginning of the first α-helix in the C-terminal domain of the MSA and MSG isoforms: Ser 342, Gly 417, and Glu 595 in hvMSH, ecMSA and ecMSG respectively. Again, the direction of the trajectory of the preceding segment in hvMSH is quite different from those of MSA and MSG, essentially orthogonal (Figure 5b). The connection between the last common structure in the TIM barrel, and that of the first common structure in the C-terminal domain among these three isoforms consists of 69 residues in hvMSH, 36 in ecMSA and 45 in ecMSG. Of these 69 residues in hvMSH, 43 are disordered in the crystal structure, preventing a more detailed comparison for this region.

The overlays reveal that the C-terminal domain of hvMSH, which caps the active site of the TIM barrel, is quite different from those found in ecMSA and ecMSG. This C-terminal domain, consisting largely of a bundle of five α-helices, is closely related in MSA and MSG. However, only two of these α-helices are structurally conserved in hvMSH, connected by a β-hairpin which is also conserved among all three isoforms (Figure 5b). While the β-hairpin is conserved, the length of each hairpin varies substantially, with that of hvMSH fifteen residues longer and ecMSG five residues longer than the hairpin in ecMSA. Only the two ends of each β-strand at the base of the hairpin superimpose closely in all three structures, along with the C-terminal end of the preceding α-helix (helix 1), and the N-terminal end of the following helix (helix 2) (Figure 5b). This close structural alignment is an important region of the C-terminal domain since it contributes the catalytic base to the active site. This catalytic base, Asp 388 in hvMSH, resides at the junction between the second strand of the β-hairpin, and helix 2 of the C-terminal domain. The backbone carbonyl of the preceding residue, Trp 387, is involved in the last H-bond in the β-hairpin, while the backbone carbonyl of Asp 388 accepts the first H-bond of the helix. It is in a position which might be expected to cap this helix, but the backbone geometry prevents it from forming an H-bond to the amide NH (N-O distances are 3.6, 3.8 and 3.7 Å for hvMSH, ecMSA and ecMSG respectively). While the N-terminus of this helix aligns fairly well in all three structures, they eventually diverge at the C-terminal end with the helix in hvMSH having a drastic bend in the middle due to Pro 398. Helix 2 in ecMSA is also slightly bent although no proline residues are present, but is not bent in ecMSG. A comparison of the remaining segment of each protein following their roughly common departure point from this helix, shows that while ecMSA and ecMSG are quite similar, the structure adopted by the C-terminal residues of hvMSH is radically different and is also significantly shorter (Figure 5c). This final segment of the protein in hvMSH is 41 residues shorter than ecMSA, and 47 residues shorter than ecMSG, contributing to its smaller overall size.

### Evolutionary implications

**Figure 6:**
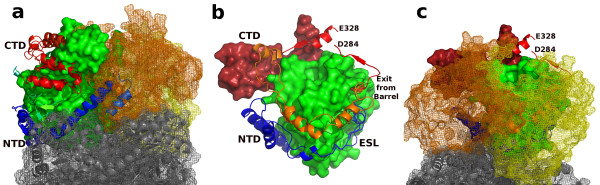
**Relationship of hvMSH oligomerization interfaces to N-terminal clasps and connecting segments in MSA and MSG**. Superposition of hvMSH, ecMSA and ecMSG ternary complexes using SSM in Coot. a) N-terminal domain. MSA and MSG are shown as cartoon ribbon traces with N-terminal (NTD) and C-terminal (CTD) domains colored blue and red respectively. Only portions of the CTD which are not homologous to hvMSH are visible. MSH is shown as a green solvent-accessible surface. Other MSH subunits which make up a trimeric assembly are rendered as copper and yellow mesh surfaces; the subunits of the symmetry-related trimer which forms the hexameric assembly are colored grey and shown as mesh surfaces surrounding cartoon ribbon traces. b) Locations of connecting loops; view is from the right side of part a. MSH is shown as a molecular surface with the barrel domain and C-terminal domain colored green and red respectively. Extended surface loops (ESL; blue) connecting the NTD to barrel domains, and loops (orange) connecting barrel domains to the CTD in MSA and MSG are shown as cartoon ribbon traces. The segments of the connection between the barrel domain and CTD of MSH which are visible in electron density maps are depicted as a red cartoon ribbon trace. c) View identical to b, but including other subunits of the trimeric and hexameric hvMSH assembly colored and rendered as in part a.

Comparisons of the oligomeric structure of hvMSH with the previously determined monomeric structures of ecMSA and ecMSG highlight structural differences described above, and provide potential insight into their evolutionary relationships. The N-terminal domain and extended surface loop preceding the TIM barrel domain in MSA and MSG, are absent in MSH. The surface of the barrel interacting with these N-terminal sequences in ecMSA and ecMSG is instead largely covered by oligomerization interfaces of the trimer and hexamer in hvMSH (Figure 6). Additionally, the segments which connect the barrel domain to the C-terminal domain in these three isoforms interact with completely different parts of the barrel surface in hvMSH versus ecMSA and ecMSG as they travel from one end of the barrel to the C-terminal domain which caps the active site at the opposite end (Figure 6b). The paths taken by these connecting segments in ecMSA and ecMSG run roughly parallel to those of the extended surface loops which connect the N-terminal and barrel domains. The surface of the barrel covered by both of these connecting segments in either ecMSA or ecMSG is instead covered by neighboring subunits in the formation of the hvMSH trimer (Figure 6b, c).

These observations suggest the possibility that the N-terminal deletion in MSH and oligomerization are related. One possible scenario would be that an ancestral monomeric enzyme acquired mutations that destabilized the interactions between the N-terminal sequences and barrel and at the same time favored a weak intersubunit aggregation. A displaced N-terminal domain would have then become expendable since exposed regions of the barrel surface would be buried and any potentially stabilizing effects to the enzyme structure could have been satisfied by interactions with neighboring subunits. These interactions, fine-tuned by natural selection, would then allow for a functional, soluble enzyme in the event of an N-terminal deletion in the gene. Of course, this is only one possible scenario, and the reverse can also be imagined where an oligomeric enzyme acquired an N-terminal extension which was able to compete for and replace subunit interactions to become a stable monomer. Regardless of the actual process involved, the structural comparisons are consistent with an evolutionary model in which N-terminal deletion and oligomerization are coupled. It will be interesting to see future structural determinations of oligomeric forms of MSA, which presumably still have N-terminal domains yet form stable multimers [43-45], to understand how they have adapted to interact with neighboring subunits.

### Comparison of the active site of *H. volcanii *MSH with those of *E. coli *MSA and MSG

**Figure 7:**
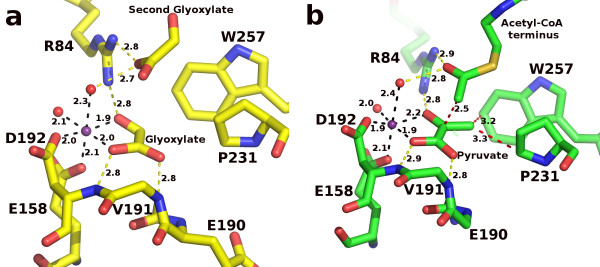
**The active sites of *H. volcanii *MSH**. a) High-occupancy glyoxylate complex rendered in stick form with carbon atoms yellow, oxygen red, and nitrogen blue. The magnesium ion and coordinating water molecules are shown as spheres in purple and red respectively. Distances are shown in angstroms with hydrogen bonds yellow and metal-ligand bonds in black. The side chain of Val 191 has been removed for clarity. b) Pyruvate/Acetyl-CoA ternary complex rendered as in part a, but with carbon atoms in green and sulfur in tan. Additionally, close contacts are indicated with red dashed lines. Side chains for Glu 190 and Val 191 have been omitted for clarity.

**Figure 8:**
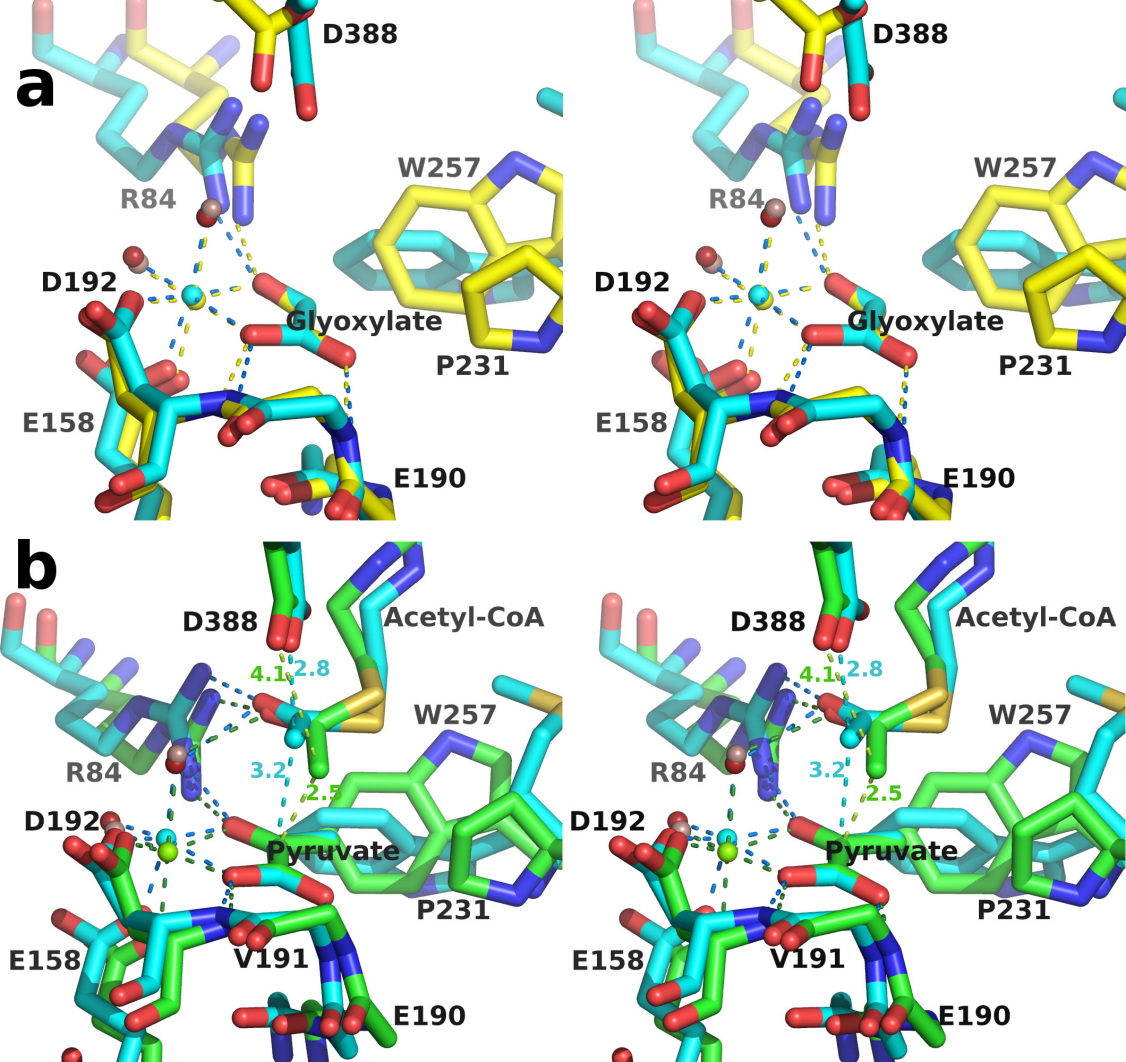
**Overlays of the *H. volcanii *MSH active sites with the corresponding *E. coli *MSG complexes**. Overlays were performed by superimposing glyoxylate molecules in part a, or pyruvate molecules in part b, using LSQ in Coot. Residue numbers refer to the *H. volcanii *sequence. Side chains at positions 190 and 191, and at corresponding positions in ecMSG, have been omitted for clarity. a) Stereoview of high-occupancy glyoxylate complex overlay [PDB:1D8C for ecMSG]. Rendered as in figure 7a, with hvMSH carbon atoms yellow, and ecMSG carbons blue. The magnesium ion and water molecules are colored blue and red respectively in the ecMSG complex, and yellow and lilac in the hvMSH complex. All hydrogen and metal-ligand bonds are colored yellow in hvMSH, and blue in ecMSG. b) Stereoview of pyruvate/Acetyl-CoA ternary complex overlay [PDB:1P7T for ecMSG]. Rendered as in part a, but with carbons, magnesium ions, and bonding interactions in green or blue for hvMSH or ecMSG respectively. Water molecules are lilac for hvMSH and red for ecMSG. Distances are shown in angstroms to show proximity of the acetyl-CoA methyl carbon to either the catalytic base oxygen (Asp 388) or the ketone carbonyl of pyruvate.

The active site of hvMSH is very similar to those of ecMSA and ecMSG. Figure 7 shows the active site of hvMSH in the two complexes reported here. Figure 8 shows overlays of the active site region of hvMSH with the corresponding complexes of ecMSG. Overlays were performed by least squares superposition of the glyoxylate or pyruvate molecule in each complex using the LSQ algorithm in Coot [46]. Unfortunately examples of MSA in complex with glyoxylate or pyruvate are not available for detailed comparisons, but the active sites of MSA and MSG are very similar with identical catalytic groups in identical conformations [4]. The glyoxylate binding determinants are the same in both hvMSH and ecMSG [PDB:1D8C] [34], with the carboxylate group of glyoxylate accepting two main chain hydrogen bonds from consecutive residues at the N-terminus of an α-helix (Val 191 and Asp 192 in hvMSH; Leu 454 and Asp 455 in ecMSG), and one oxygen coordinating a bound magnesium ion. The aldehyde oxygen of glyoxylate forms a second bond to the magnesium ion as well as a hydrogen bond to Arg 84 (Arg 338 in ecMSG) (Figure 7a, 8a). The enzyme also coordinates the magnesium ion with identical residues to those found in MSG and MSA: the side chains of Asp 192 and Glu 158 in hvMSH (Asp 455 and Glu 427 in ecMSG). Two water molecules complete the fifth and sixth positions in the magnesium coordination sphere with nearly perfect octahedral geometry as is seen in ecMSG. One notable difference in the active site between hvMSH and ecMSG is the conformation of the tryptophan residue adjacent to the aldehyde carbon of glyoxylate (Trp 257, and Trp 534 in hvMSH and ecMSG respectively) (Figure 8a). The rotamer in the ecMSG complex places the edge of the indole ring 4.0 Å from the glyoxylate aldehyde carbon. However, in the hvMSH complex the different rotomer positions the indole ring to interact more with its face than edge, with distances to the two closest carbons in the ring of 3.7 and 3.8 Å. The rotomer in the hvMSH structure is held in position by the side chain of Phe 14 which is packed against the opposite side of the indole ring. The structurally equivalent position in ecMSG is Gln 116, which forms a hydrogen bond to the indole NH group to stabilize the more edge-on interaction.

**Figure 9:**
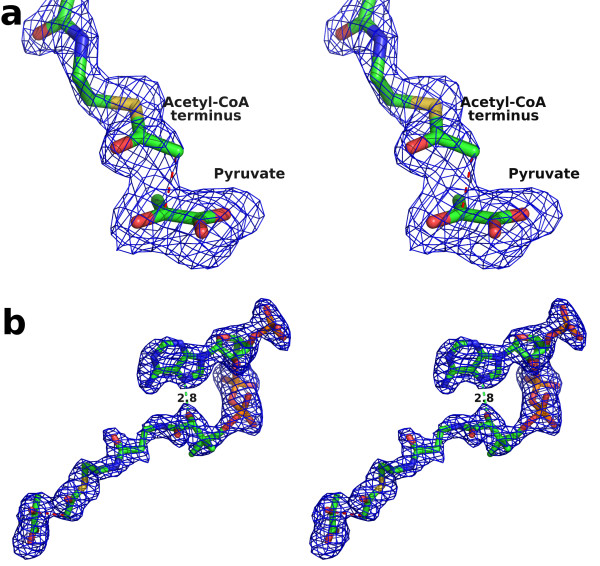
**Acetyl-CoA/pyruvate omit map showing close contact and the bent conformation of Acetyl-CoA**. An F_o_-F_c _electron density omit map contoured at 3 σ is superimposed on the the final refined model of acetyl-CoA and pyruvate in stick form. Ten rounds of refinement were performed on a model with both molecules removed, prior to map calculation. a) Stereo view of difference density showing the ~2.5 Å close contact (red dashed line). b) View of the map in the region of the active site and acetyl-CoA binding site. An intramolecular hydrogen bond also observed in the ecMSG ternary complex is shown as a green dashed line.

The overlay of the ternary complexes of hvMSH and ecMSG [PDB:1P7T] [36] shows a very similar configuration of active site residues and binding interactions with pyruvate to those seen in the glyoxylate complexes (Figure 7b, 8b), however, the position of the acetyl moiety of acetyl-CoA is unique to hvMSH. In the ecMSG ternary complex, the methyl carbon of the acetyl group makes a ~2.8 Å unfavorable contact with the side-chain carboxylate of the presumed catalytic base (Asp 631) that is closer than the sum of the van der Waals radii. This was interpreted to represent the active site geometry for proton abstraction from the terminal methyl group of acetyl-CoA, similar to cases observed in citrate synthase [36]. In the hvMSH complex, however, the position of this terminal acetyl group is quite different. While in both cases, the acetyl group carbonyl oxygen forms hydrogen bonds to the conserved arginine (Arg 84 in hvMSH and Arg 338 in ecMSG), and an axial water molecule in the magnesium coordination sphere, the terminal methyl carbon is instead making an unfavorable contact (~2.5 Å) with the carbonyl carbon of the pyruvate keto group (Figure 8b). This terminal acetyl group is in a conformation which appears to correspond to a nucleophilic attack on pyruvate, but is unable to complete the formation of a covalent bond (See discussion of the catalytic mechanism below). The evidence for this close contact is clearly seen in an omit map for pyruvate and acetyl-CoA contoured at 3 σ (Figure 9a). Refinement trials in which restraints for non-bonded contacts were increased in an attempt to increase this unfavorable contact distance simply resulted in the atoms being pushed out of the 2F_o_-F_c _electron density, with a simultaneous formation of a positive difference peak between the methyl group and pyruvate keto group in F_o_-F_c _maps, leading us to conclude that this refined distance is real.

### Acetyl-CoA binding site

The acetyl-CoA binding sites in MSH, MSA and MSG are located at structurally equivalent positions. Acetyl-CoA binds to hvMSH in a bent conformation similar to that seen in the ecMSG ternary complex (Figure 9b). In both cases an intramolecular hydrogen bond forms between adenine N7 and the hydroxyl group of the pantothenate moiety. There are also two hydrogen bonds between the exocyclic N6 of the adenine ring and two backbone carbonyls that are structurally conserved in all three isoforms. One of these carbonyls in the hvMSH structure also forms a hydrogen bond (3.0 Å O-N bond distance) to the amide NH of the pantothenate moiety of the acetyl-CoA. The comparable interaction is not observed in the ecMSG ternary complex (3.7 Å O-N distance). Unfortunately the pantothenate, β-mercaptoethylamine and acetyl portions of acetyl-CoA were not visible in the ecMSA ternary complex [4], precluding a direct comparison in these regions of the acetyl-CoA binding site. Additionally, there is a hydrogen bond between adenine N1 and the side chain hydroxyl of Ser 17 in the hvMSH structure which is absent in both the ecMSA and ecMSG adenine binding sites. The structurally equivalent positions are Gly 96 and Val 119 in ecMSA and ecMSG respectively. The adenine ring binds in a hydrophobic pocket against a helix-capping proline as seen in both ecMSA and ecMSG (Pro 261, Pro 369, and Pro 538 in hvMSH, ecMSA and ecMSG respectively). Adjacent to Pro 261, the side chain of Phe15 contributes to the hydrophobic pocket on the same side of the adenine ring. The structurally equivalent position is Ile 94 in ecMSA and Leu 117 in ecMSG. Met 30 packs against the opposite side of the adenine ring, and is structurally equivalent to Met 102 in ecMSA and Tyr 126 in ecMSG. The terminal carbonyl of the pantothenate moiety forms a hydrogen bond to the side chain of Thr 16 in HvMSH. This same position in ecMSA is Thr 95 and therefore may form a similar interaction, but is Val 118 in ecMSG. Met 508 of ecMSG (Met 330 in ecMSA), which forms a hydrophobic interaction for the β-mercaptoethylamine portion of acetyl-CoA, is replaced by Pro 231 in hvMSH, which also interacts with the methyl group of the pyruvate molecule bound in the active site. The hydrophobic surface formed by Met 508 in ecMSG is partially formed by Leu 259 in the hvMSH structure.

Cys 617 in ecMSG and Cys 438 in ecMSA have both been observed to be oxidized to sulfenic acid in crystal structures of these enzymes, suggesting a potential catalytic and/or regulatory function [4,36]. The equivalent position in hvMSH is Val 119, with no cysteine residues occurring in the active site. There is only one cysteine residue in hvMSH (Cys 225), which is located on the opposite end of the TIM barrel from the active site. Even this single cysteine is not conserved among MSH isoform members, apparently eliminating the possibility of a potentially similar type of redox regulatory function in this isoform.

### Structural Overlay of HvMSH glyoxylate and ternary complexes

**Figure 10:**
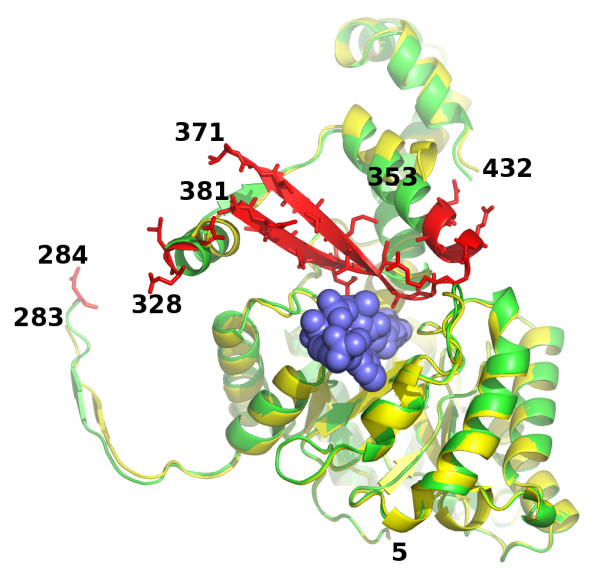
**Overall structural comparison of the two *H. volcanii *complexes**. The two models were superimposed using SSM in Coot. The high-occupancy glyoxylate complex is shown in yellow and the corresponding sequences of the ternary complex in green. Portions of the enzyme that become ordered upon acetyl-CoA binding in the ternary complex are colored red and also depicted in stick form. Acetyl-CoA is colored slate blue in space-filling form.

**Figure 11:**
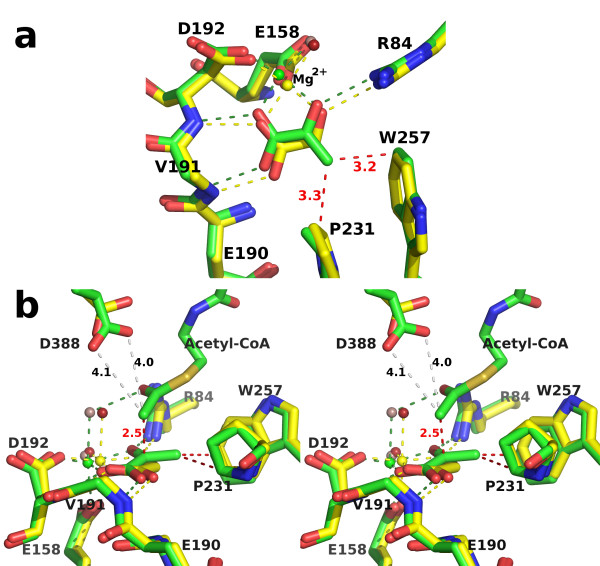
**Structural comparison of the active sites of the two *H. volcanii *complexes**. SSM overlay as in figure 10, but showing detail in the region of the active site. The side chain of Val 191 has been omitted for clarity. a) Top view, roughly perpendicular to the glyoxylate molecule. Carbon atoms, magnesium ions, hydrogen and metal-ligand bonds are colored yellow or green in the glyoxylate or ternary complex respectively. Water molecules are depicted as spheres colored red in the glyoxylate and lilac in the ternary complex. Close contacts to the pyruvate methyl group are shown as red dashed lines with distances in angstroms. b) Side view of part a in stereo. Additionally, the close contact between the acetyl methyl carbon of acetyl-CoA and the ketone carbonyl carbon in pyruvate, and the distances between this methyl carbon and the side chain carboxylate oxygens of Asp 388 are shown in red and grey dashed lines respectively.

A superposition of the two *H. volcanii *MSH complexes we report here, using SSM in Coot, reveals portions of the enzyme that become ordered in the pyruvate/acetyl-CoA complex (Figure 10). Within the C-terminal domain, most of the β-hairpin and the C-terminal half of the preceding helix, fold in over the top of the bound acetyl-CoA to complete its binding site. When the active sites are compared in detail, however, additional differences become apparent (Figure 11). The most obvious is the movement of Asp 388, the presumed catalytic base, down into the active site (0.8 and 1.4 Å shift for the two carboxylate oxygen atoms). More subtle shifts are noticeable from a 'top' view, roughly perpendicular to the plane of the glyoxylate molecule (Figure 11a). All of the ligands which coordinate the carboxylate and aldehyde groups of glyoxylate are shifted in the direction of the magnesium ion upon pyruvate and acetyl-CoA binding, with the magnesium ion shifting by 0.56 Å. At the same time, the methyl group of pyruvate forms two close contacts with Pro 231 and Trp 257, apparently pushing these two residues apart, with the distance between the proline gamma carbon and the indole ring increasing by ~0.5 Å. The amide nitrogen atoms which form hydrogen bonds with the carboxylate of pyruvate are shifted away from pyruvate by 0.35 and 0.18 Å relative to their positions in the glyoxylate complex. Looking from a side view reveals that the pyruvate molecule is not bound in the same plane which accommodates the glyoxylate, a position which would be expected to represent an ideal geometry for catalytic turnover (Figure 11b). Instead the close contacts of the methyl group with Pro 231 and Trp 257 appear to prevent the pyruvate from dropping fully into the binding site, despite the spreading apart of the active site in the ternary complex. It thus appears that pyruvate, in the ternary complex, has forced the active site to spread apart relative to the glyoxylate complex but has reached its limit.

### Catalytic Mechanism

A plausible catalytic mechanism for malate synthase was first proposed based on the crystal structure of the glyoxylate complex of MSG from *E. coli *[PDB:1D8C] [34], with Asp 631 acting as a catalytic base to deprotonate the methyl group of acetyl-CoA to form an enol(ate) intermediate stabilized by Arg 338 (corresponding residues in hvMSH are Asp 388 and Arg 84). The enol(ate) was proposed to swing down to attack the aldehyde carbon of glyoxylate to form the malyl-CoA intermediate which is subsequently hydrolyzed. This mechanism is consistent with the observed inversion of configuration of the acetyl-CoA methyl group during the reaction [47,48], and has been supported by subsequent crystal structures of malate synthases in complex with substrates and inhibitors [4,35-37]. Additionally, site directed mutagenesis has confirmed the importance of both Asp 631 and Arg 338 for catalytic activity [36]. Asp 631 was shown to be absolutely essential, with a D631N mutation rendering the enzyme activity unmeasurable, while Arg 338 could be replaced by lysine with activity reduced to 6.6% of wild type. The structures reported here are also consistent with this proposed mechanism, having identical catalytic and magnesium-coordinating residues observed in all previously determined malate synthase structures. While the structure of the active site of the glyoxylate complex reported here is very similar to other previously determined glyoxylate complexes [34,35], the structure of the hvMSH ternary complex appears to add a novel observation addressing the catalytic mechanism. There is only one previously determined malate synthase structure in which the terminal region of acetyl-CoA has been seen in electron density maps, to allow the position of the acetyl group in the active site to be identified: the *E. coli *MSG ternary complex [36]. In this structure [PDB:1P7T] the terminal methyl group of acetyl-CoA is making a close contact with the proposed catalytic base Asp 631, refining to a C-O distance of 2.78 Å. The distance from this methyl carbon to the ketone carbonyl carbon of pyruvate refined to 3.16 Å. The close contact with the catalytic base supports the proposal that Asp 631 acts to deprotonate the terminal methyl group in the enolization step of the reaction. In the ternary complex reported here for hvMSH, however, the acetyl group is seen to bind in a different relative position in the active site. The distance between the carboxylate oxygen of the catalytic aspartate (Asp 631 and Asp 388, in ecMSG and hvMSH respectively) and the ketone carbonyl carbon of pyruvate is similar in both complexes: ~5.9 Å and ~6.2 Å respectively for ecMSG and hvMSH. But rather than forming a close contact with the catalytic base as seen in the ecMSG ternary complex, the terminal methyl carbon of acetyl-CoA instead forms a close contact (2.46 Å refined distance) with the electrophilic keto carbon in pyruvate, and is ~4.1 Å from the reactive oxygen of the catalytic base (Figure 8b, 11b). It appears that the hvMSH ternary complex is well along the reaction coordinate of a nucleophilic attack on pyruvate, with the contact distance intermediate between that of a van der Waals interaction and a covalent bond.

Enolization of acetyl-CoA has been demonstrated for yeast malate synthase which, in the presence of pyruvate, catalyzes isotopic exchange between acetyl-CoA and tritiated water [44,49]. Therefore, the enol(ate) form of acetyl-CoA is expected to exist at least transiently in the ternary complex. But if the structure does indeed show the enolate intermediate in the process of bond formation with the carbonyl carbon, why is it arrested along the reaction pathway? Pyruvate, while able to stimulate the enolization of acetyl-CoA, is in fact an inhibitor of malate synthase, unable to complete the reaction [49]. The forced expansion of the active site described in the previous section and the close contacts the methyl group of pyruvate makes with Pro 231 and Trp 257 appear to prevent the formation of the tetrahedral geometry required for the condensation reaction. Whereas there is plenty of space for a hydrogen atom attached to the electrophilic carbonyl in glyoxylate to drop below the plane to form the tetrahedral transition state, the methyl group appears constrained. This is analogous to the situation seen in complexes of bovine pancreatic trypsin inhibitor and trypsin where the active site serine oxygen makes a close contact (~2.6 Å) to the peptide carbonyl carbon of BPTI, but is prevented from completely reacting by the constraints imposed by the enzyme and inhibitor, thus freezing the process at an intermediate state of the nucleophilic addition reaction [50,51]. Similar reaction intermediates interpreted as nucleophilic addition reactions proceeding to varying extents have been observed in small molecule crystal structures containing nucleophilic nitrogen atoms and electrophilic carbonyl groups, with nitrogen-carbonyl carbon distances ranging from 2.9 to 1.5 Å [52]. As in the analysis of the trypsin/protein inhibitor complexes, these cases were interpreted to arise from the constraints imposed by the crystal environment which froze the addition reaction at intermediate points along the reaction coordinate. An analysis of these structures led to insight into the reaction pathway that was confirmed by theoretical calculations and improved understanding of the process [53]. Thus, we interpret the close contact in our ternary complex to represent the enolate intermediate of acetyl-CoA caught in the process of bond formation with the carbonyl carbon of pyruvate, but unable to complete the process due to steric hindrance. This implies that removal of atoms responsible for the steric hindrance would allow the reaction to proceed to completion. Therefore, we would expect the double mutant W257H, P231A, if still folding competent, to acquire the ability to catalyze acetyl transfer from acetyl-CoA to pyruvate.

### Halophilic Adaptation

As expected, hvMSH exhibits characteristics similar to those seen in other halophilic proteins. It has a marked increase in acidic amino acids with 95 of the 433 residues being either glutamic or aspartic acid making the protein 21.9% acidic. This is consistent with other halophilic proteins [26], however hvMSH contains a greater amount of glutamic acid residues (55) than aspartic acid residues (40). By comparison, ecMSA and ecMSG are 13.5% and 12.3% acidic respectively. Utilizing PISA (Protein Interactions, Surface, and Assembly) [40] to analyze the trimeric assembly, it was determined that of the 78 acidic residues per subunit that are ordered in the ternary complex, 41 are solvent accessible, 35 are buried at intersubunit interfaces, and two are inaccessible, making ~52% of all ordered acidic residues accessible to solvent. Of the 159 total residues in each monomer accessible to solvent in the trimeric assembly of hvMSH, 25.8% are acidic. The single cysteine and the nine lysine residues found in hvMSH are also consistent with what is seen in proteome surveys of halophilic organisms, which show that halophilic proteins have an underrepresentation of cysteine and lysine [26]. The number of expected cysteine and lysine residues for a protein of this size, based on the average occurance typically found in proteins (1.9 and 5.9% respectively) [54] would be approximately 8 and 25.

*H. volcanii *MSH also demonstrates a substantial number of intermolecular ion pairs. An analysis of the three different protein interfaces present in the trimeric and hexameric assemblies showed that the interface between monomers of the trimer contains six intermolecular salt bridges. Of the two interfaces per subunit between the two trimers in the hexameric assembly, one has no salt bridges, while the other has eight. Thus the total number of intermolecular salt bridges stabilizing the trimer is 18 (six at each interface). The hexamer is stabilized by an additional 24 intermolecular salt bridges (eight at each pair of subunits across the interface) for a total of 60 in the hexameric assembly. *H. volcanii *MSH also is seen to bind a number of solvent ions: three potassium and 4 or 5 chloride ions per subunit in the pyruvate/acetyl-CoA ternary complex and the high-occupancy glyoxylate complex respectively, with one K^+ ^and one Cl^- ^ion bound at the trimer interface. Interestingly, the ternary complex binds a phosphate ion along the three-fold axis of the trimer at the same position of the fifth chloride ion that is observed in the glyoxylate complex.

### Sequence analysis in light of the structure

**Figure 12:**
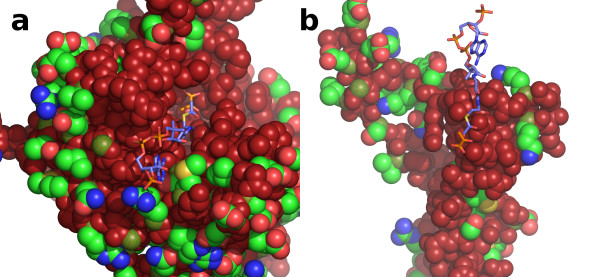
**Strictly conserved residues among all nine homologous sequences in table 2**. a) view looking into the active site of the TIM barrel domain, with the C-terminal domain removed for clarity. The surface is shown in space filling form with main chain peptides and strictly conserved residues colored brick red, and side chains of non-conserved residues colored according to atom type with carbon atoms green. The Cα atoms of nonconserved residues are colored green stippled with red. Acetyl-CoA and pyruvate are depicted in stick form showing their binding locations in the ternary complex with carbon atoms colored slate blue or orange respectively. b) same as in part a, but showing C-terminal domain interactions.

A basic local alignment search using the BLAST tool at the Universal Protein Resource [9] with *H. volcanii *MSH (*aceB *gene, [UniProt:Q977U4]) as the query sequence reveals eight other similar protein sequences with identities ranging from 73 and 99 percent (Table 2). In addition to this high level of sequence identity, the sizes of these proteins are also close, ranging from 433 to 441 amino acid residues in length. Despite this level of similarity, only three of these eight have been annotated as malate synthase enzymes. Two are encoded by genes in *H. volcanii *[16][UniProt:D4GTL2 and D4GPK1] (encoded by the *aceB1 *and *aceB2 *genes respectively), the first of which is the same length as the query sequence and differs by only a single amino acid which resides in one of the disordered loops in the crystal structures reported here. Based on its relative position to the single isocitrate lyase gene in the *H. volcanii *genome [16,55], the aceB and aceB1 genes appear to be the same, differing by a single nucleotide polymorphism between the two strains involved (DSM 3757 and DS2 respectively). The second is three residues longer and only 78% identical. A check of the electron density at sites that differ in sequence shows that the enzyme we have purified and crystallized from the native *H. volcanii *strain DS2 is the *aceB1 *gene product. The only other sequence in table 2 annotated as malate synthase is found in *Haloarcula marismortui*, and is two residues longer than the query and 81% identical in sequence [17]. Interestingly, this protein was recently shown to be bifunctional, catalyzing the malate synthase reaction in two steps and functioning as an (S)-malyl-CoA lyase/thioesterase. Reclassified as an "apparent malate synthase", it functions in a recently postulated methylaspartate cycle for acetyl-CoA assimilation in *H. marismortui*, rather than a glyoxylate cycle as occurs in *H. volcanii *[56]. The five other sequences listed in table 2, however, have been annotated as enzymes other than malate synthase: the citrate lyase beta subunit from *Halogeometricum borinquense *[57], the HpcH/Hpal aldolase from *Haloterrigena turkmenica *[UniProt:D2S276], the citryl-CoA lyase from *Haloquadratum walsbyi *[32], the Homolog to citryl-CoA lyase from *Natronomonas pharaonis *[58] and the HpcH/HpaI aldolase from *Natrialba magadii *[UniProt:D3SSR8]. The high sequence identities and similar sizes prompted a further investigation into these five proteins. An alignment using ClustalW [59] reveals that approximately 50% of residues (220) were strictly conserved in all nine sequences. An analysis of the positions of these strictly conserved residues shows that all residues in the active site including the magnesium coordinating ligands, catalytic acid and base, and all residues in the acetyl-CoA binding site with the exception of three near the adenosine moiety are strictly conserved (Figure 12). One of these three, Ser 17 forms a hydrogen bond to the N1 of the adenine ring and another to the epsilon nitrogen of Trp 46. This residue is conservatively replaced by threonine in two of the nine sequences, which would also be able to satisfy both of these hydrogen bonds. The second nonconserved residue is Met 30 which is replaced by lysine in one of the nine sequences. Met 30 forms one side of the hydrophobic binding pocket for the adenine ring. A lysine at this position could presumably fulfill the role of Met 30 with the four methylene carbons of its alkyl chain which could adopt a sterically similar structure. Both the sulfur and terminal methyl group of Met 30 are solvent exposed which would allow the terminal ammonium group on a lysine at this position to be stabilized by water or ions in solution. The final residue which is not strictly conserved is Arg 33 which makes a salt bridge to the 3'-phosphoryl moiety of acetyl-CoA. It is replaced by serine in five of the nine sequences which would not be able to fulfill a similar role. However, since all of the nine sequences in table 2 are found in halophilic organisms, functioning in high ionic-strength conditions, the solvent exposed 3'-phosphate would presumably be stabilized by positively charged ions in solution. Since all the residues involved in the catalytic mechanism are strictly conserved, and all residues involved in binding interactions with substrates are strictly conserved, or appear to remain functional in the three exceptions just discussed, it appears that these five sequences may have been misannotated and are in fact members of the malate synthase H isoform family.

**Table 2:** Protein sequences closely related to *H. volcanii *MSH [UniProt:Q977U4]

UniProt Accession	Protein Name	Organism	Length	Identity
* Q977U4	Malate Synthase	*Halobacterium volcanii (Haloferax volcanii)*	433	100%

* D4GTL2	Malate Synthase	*Haloferax volcanii *(strain ATCC 29605/DSM 3757/IFO 14742/NCIMB 2012/DS2)	433	99%

Q5V0X0	Malate Synthase	*Haloarcula marismortui (Halobacterium marismortui)*	435	81%

E4NU70	Citrate Lyase Beta Subunit	*Halogeometricum borinquense (strain ATCC 700274/DSM 11551/JCM 10706/PR3)*	434	80%

D2S276	HpcH/HpaI aldolase	*Haloterrigena turkmenica *(strain ATCC 51198/DSM 5511/NCIMB 13204/VKM B-1734) (*Halococcus turkmenicus*)	436	78%

D4GPK1	Malate Synthase	*Haloferax volcanii *(strain ATCC 29605/DSM 3757/IFO 14742/NCIMB 2012/DS2)	436	78%

Q18JF9	Citryl-CoA lyase	*Haloquadratum walsbyi *(strain DSM 16790)	435	78%

Q3INJ7	Homolog to citryl-CoA lyase	*Natronomonas pharaonis *(strain DSM 2160/ATCC 35678)	436	76%

D3SSR8	HpcH/HpaI aldolase	*Natrialba magadii *(strain ATCC 43099/DSM 3394/NCIMB 2190/MS3) (*Natronobacterium magadii)*	441	73%

* Q977U4 and D4GTL2 correspond to the same gene with a single nucleotide polymorphism (See text).

## Conclusions

The structures reported here for the glyoxylate and the pyruvate/acetyl-CoA complexes of *Haloferax volcanii *malate synthase represent the first examples of an H isoform member. Instead of the expected tetramer [15], a trimer is found to be the major state in solution, although an equilibrium with a significant hexamer population is evident.

The overall structure of hvMSH reveals that, like MSA and MSG, this halophilic isoform is based on a TIM barrel and indicates that deletion in hvMSH of an N-terminal domain distinguishes this isoform from those of MSA and MSG; and that the surface of the barrel normally buried by this domain and connecting loops is instead involved in trimeric and hexameric interfaces, suggesting a potential evolutionary coupling of the N-terminal deletion and oligomerization.

Despite the sequence divergence and overall smaller size of hvMSH compared to MSA and MSG, the active site and catalytic mechanism are conserved in all three isoforms. In the ternary complex of hvMSH, however, the position of the terminal methyl group of acetyl-CoA is found to differ considerably from that seen in the ecMSG ternary complex. Instead of a structure corresponding to the deprotonation step by the catalytic aspartate as seen in ecMSG [36], the ternary complex of hvMSH reveals this methyl group interacting closely with the carbon atom of the electrophilic carbonyl of pyruvate, in an apparent nucleophilic attack arrested by steric hindrance. Therefore, the ternary complexes of ecMSG and hvMSH are complementary, revealing the active site configurations for two important steps in the catalytic mechanism: proton abstraction by the catalytic base, and nucleophilic attack of the enolate intermediate on the electrophilic substrate.

## Methods

### Protein isolation

*Haloferax volcanii *Malate synthase encoded by the *aceB1 *gene was produced and purified as previously described [10,18]. Briefly, a lyophilized sample of *Haloferax volcanii *was obtained from the American Type Culture Collection [60], and grown at 37°C in a chemically-defined medium with acetate as a sole carbon source to induce expression of glyoxylate cycle enzymes. Cells were lysed by sonication on ice. Protein purification was performed at 4°C using three chromatographic steps: reverse phase, anion-exchange and gel-filtration as previously described [18]. Calibration of the Sephacryl-300 sizing column (Pharmacia) was performed with gel-filtration standards from Bio-Rad: Thyroglobulin (bovine), 670 kDa; γ-globulin (bovine), 158 kDa; Ovalbumin (chicken), 44 kDa; Myoglobin (horse), 17 kDa; and Vitamin B_12_, 1.35 kDa. Progress was monitored by silver-stained SDS PAGE analysis and enzyme activity assays. Average yield was 0.5 mg of ~90% pure enzyme per liter of cell culture.

### Enzyme Activity Assay

Malate synthase activity was measured by monitoring the loss of absorbance at 232 nm upon acetyl-CoA thioester cleavage as previously described [18,49]. The reaction conditions were 0.34 mM acetyl-CoA, 1.1 mM glyoxylate, 20 mM Tris pH 8.0, 2 mM EDTA, 3 M KCl, and 5 mM MgCl_2_. The reaction was initiated by the addition of 10 μL of enzyme solution into a 1 mL total reaction volume.

### Crystallization, and heavy atom derivatization

Crystals of *H. volcanii *MSH were grown at room temperature in sitting drops as previously described [18]. The protein solution contained *H. volcanii *malate synthase at 7 mg/mL, 13 mM MgCl_2_, 3 mM glyoxylate, 50 mM Tris·HCl pH 8.0, and 2 M KCl. The well solution contained 0.17 M ammonium acetate, 24.5-27% w/v PEG 4500, 15% glycerol, and 0.085 M sodium acetate trihydrate at a pH of 4.4-5.0. Two microliters of protein solution were mixed with an equal volume of well solution, and allowed to equilibrate at room temperature. Crystals grew over a period of approximately two weeks. A lead derivative was prepared by addition of 0.4 μl of a saturated lead (II) acetate solution to an equilibrated drop after crystal growth was complete. A high-occupancy glyoxylate complex was produced by increasing the concentrations of MgCl_2 _and glyoxylate to ~0.1 M in drops of mother liquor containing fully grown crystals. The ternary complex of pyruvate and acetyl-CoA was produced using the same well solution as above, and a protein solution containing *H. volcanii *malate synthase at 7 mg/mL, 50 mM MgCl_2_, 50 mM Tris·HCl pH 8.0, and 2 M KCl. Pyruvate and acetyl-CoA were added to equilibrated drops of mother liquor following crystal growth to ~70 mM and ~0.15 M respectively.

### Data collection, processing, phasing and structure determination

Crystals were suspended in nylon loops and cryocooled by plunging into liquid nitrogen. Data were collected at 100 K on an R-axis IV detector using Copper Kα radiation produced by a Rigaku 007 HF rotating anode generator equipped with Osmic confocal X-ray optics. Data were indexed, integrated and scaled with the HKL2000 package [61]. Phasing was carried out with SOLVE [62] using the single isomorphous replacement with anomalous scattering (SIRAS) method using the lead derivative data and the 2.7 Å native data (both at 3 mM glyoxylate) (Table 1), with subsequent density modification using RESOLVE [63]. Model building into the experimental map was performed manually with COOT [64] and model refinement with REFMAC5 [65,66]. High B-factors for Mg^2+ ^and glyoxylate and a distorted magnesium coordination sphere instigated a pursuit of conditions to drive a high-occupancy complex. The partially refined protein model (3 mM glyoxylate) comprising virtually all the ordered residues (6-281, 331-353, 387-432) was used for molecular replacement using PHASER [66,67] followed by cycles of manual rebuilding and refinement to solve both the high-occupancy glyoxylate complex and the pyruvate/acetyl-CoA ternary complex. The atomic coordinates and structure factors have been deposited in the PDB [68] with accession numbers 3PUG for the native structure (3 mM glyoxylate), 3OYX for the high-occupancy glyoxylate complex, and 3OYZ for the ternary pyruvate/acetyl-CoA complex.

Figures were made with PyMol (DeLano Scientific; http://www.pymol.org). Analysis of protein interfaces and buried surface area calculations were carried out with PISA [40]. Sequence alignments were conducted with ClustalW [59]. Structural alignments were performed using SSM [42] and least squares superposition (LSQ) in COOT [46,64].

## Abbreviations

ecMSA: *E. coli *malate synthase isoform A; ecMSG: *E. coli *malate synthase isoform G; hvMSH: *H. volcanii *malate synthase isoform H; PDB: Protein data bank; UniProt: Universal protein resource; SDS PAGE: Sodium dodecyl sulfate polyacrylamide gel electrophoresis; BLAST: Basic local alignment search tool; TCA: Tricarboxylic acid or citric acid cycle; TIM: Triose phosphate isomerase; CoA: Coenzyme A; MW: Molecular weight;

## Authors' contributions

BRH designed the research project. Protein isolation and enzyme assays were carried out by BRH, GCT, and KKL. Crystallization trials were performed by BRH, GCT, KKL, CDB and AMN. Data collection, processing and phasing were carried out by BRH, AMN, HLS and FGW. Model building and refinement were performed by BRH. Structural and sequence analyses were performed by BRH, CDB and AMN. The manuscript was written, and figures and tables prepared by BRH, CDB, AMN and HLS. All authors read and approved the final manuscript.
